# Segmental aneuploidies in fetuses with isolated echogenic intracardiac focus among women younger than 35 years

**DOI:** 10.1038/s41598-020-67501-9

**Published:** 2020-06-26

**Authors:** Jing Wang, Lin Chen, Li Wang, Daishu Yin, Yang Zeng, Feng Tang, Yu Tian, Hongqian Liu

**Affiliations:** 1 Department of Obstetrics and Gynecology, West China Second University Hospital of Sichuan University, Chengdu, 610041 China; 2 Key Laboratory of Birth Defects and Related Diseases of Women and Children, Ministry of Education, Sichuan University, Chengdu, 610041 China; 3 Department of Ultrasonography, West China Second University Hospital, Sichuan University, Chengdu, 610041 China

**Keywords:** Genetics, Molecular biology

## Abstract

Studies on the occurrence of segmental aneuploidoidy in fetuses with isolated echogenic intracardiac focus (EIF) are scarce. The aim of this study was to analyze whether there is an association between abnormal segmental aneuploidies and isolated EIF. This was a prospective case–control study. The study participants in the case group were fetuses that were diagnosed with isolated EIF. Samples without fetal ultrasound abnormalities but received prenatal diagnosis for other reasons (serological screening high-risk, voluntary request) were set as controls. All pregnant women were younger than 35 years old at the expected date of childbirth. Copy number variation sequencing (CNV-seq) was performed for all samples. The case group and control group successfully underwent CNV-seq analysis and exhibited 1,099 and 5,616 amniotic fluid samples, respectively. The detection rates of abnormal segmental aneuploidies in the case group and control group were 0.6% (7/1,099) and 1.1% (64/5,616), respectively; no statistically significant difference was found between the two groups (x^2^ = 2.220, P = 0.136). Isolated EIF did not increase the risk of fetal segmental aneuploidies.

## Introduction

Echogenic intracardiac focus (EIF) is thought to represent mineralization or small deposits of calcium in the heart muscle. The incidence of EIF varies between 0.86% and 30% in fetuses^1–3^. Racial difference affects the frequency of EIF in the hearts of second-trimester fetuses; Asian patients are more likely to exhibit EIF than patients of other ethnic groups^3,4^. EIF itself does not affect health or heart function; typically, EIF on prenatal sonography is absent by the third trimester.

Isolated EIF is associated with high-risk populations with a 4.8-fold increase in relative risk for trisomy 21 (T21)^5^, the likelihood ratio for T21 is significant for any marker (isolated, multiple or combined with anomaly)^6^, and the prevalence of EIF is higher in T21 than in chromosomally normal fetuses^7^. Therefore, most researchers agree that in cases without other clinical implications and a negative cell-free DNA screening, or a negative first- or second-trimester screening result, isolated EIF appears to be a benign variant and no further evaluation is required^4,8–12^. However, it is noteworthy that the studies above focused on the relationship between EIF and whole chromosome aneuploidy.

Hay et al. reported that 27.56% of clinically significant chromosomal abnormalities may be missed for patients with advanced maternal age, abnormal maternal serum screen, family history, or soft ultrasound markers including EIF^13^. Our previous studies suggested an association between pathogenic copy number variation (CNV) and fetal ultrasound soft marker; however, the samples contained fetuses with other soft markers (e.g., thickened nuchal fold, echogenic bowel, and mild ventriculomegaly)^14^. The research results of Shaffer et al. showed that clinically significant genomic alterations were identified in fetuses with soft markers (2/77, 2.6%), but there were only 6 cases with EIF in the samples, and no clinically significant genomic alterations were detected^15^. Another study from China showed that there were 3 samples with pathologic CNV in 143 fetuses with isolated EIF. However, we reanalyzed the pathogenicity of the three CNVs and found that only one CNV was pathogenic (Patient No. 2). In the other two samples with CNV, one was female carrier of Steroid sulphatase deficiency and the other was variants of uncertain significance (VOUS). In addition, the sample size of this study was small^16^. Hitherto, large-sample studies pertaining to the estimation of association between isolated EIF and segmental aneuploidie are scarce.

Although using isolated EIF as a basis for deciding to offer amniocentesis will result in more fetal losses than cases of detected T21 and will decrease the prenatal detection of fetuses with T21^17^, it is undeniable that the presence of isolated EIF increases the incidence of invasive procedures substantially^6,18^. At present, high-resolution chromosome detection techniques such as chromosomal microarray analysis (CMA) and copy number variation sequencing (CNV-seq) are widely used in fetal chromosome detection in China^19–24^. CNV-seq provide a more sensitive, accurate, and affordable approach to assess genome-wide CNVs compared with CMA^24–26^. The purpose of this study was to investigate whether isolated EIF indicates an increased risk of segmental aneuploidies and whether prenatal screening or prenatal diagnosis including segmental aneuploidies analysis should be recommended.

## Results

### CNV-seq analysis of the 1,099 fetuses with isolated EIF

CNV-seq analysis was performed successfully on amniotic fluid samples from 1,099 fetuses with isolated EIF. The mean gestational age and maternal age at the time of amniocentesis were 25.6 weeks (range 19.0–36.4) and 27.5 years (range 17.0–34.0), respectively. Among the 1,099 fetuses with isolated EIF, 763 (69.4%) fetuses had EIF in the left ventricle, 62 (5.6%) in the right ventricle, and 274 (25.0%) in both ventricles; we found 662 fetuses (60.2%) with a single EIF and 437 (39.8%) with multiple EIFs. Seven samples (0.6%) exhibited abnormal segmental aneuploidies (including pathogenic CNV (pCNV) and likely pathogenic CNV (lpCNV)), and CNVs in eight fetuses (0.7%) were classified as VOUS. All seven fetuses with abnormal segmental aneuploidies had multiple EIFs; the incidence of abnormal segmental aneuploidies in multiple EIFs was 1.6% (7/437), while no clinically significant segmental aneuploidies were found in 662 fetuses with a single EIF. The incidence of abnormal segmental aneuploidies in the fetuses with multiple EIFs was higher than those with single EIF (P-fisher = 0.002) (Table 1). Among these seven cases with abnormal segmental aneuploidies, five (71.4%) had submicroscopic CNVs (smaller than 5 Mb). The CNV-seq results of the fetuses with isolated EIF are summarized in Table 2.

**Table 1:** Distribution of the chromosomal abnormalities in the fetuses.

	Whole chromosome aneuploidy	Segmental aneuploidies				bCNV
		pCNV	lpCNV	VOUS	lbCNV	
Fetuses with EIF (n = 1,099)	1 (0.1%)	0	7 (0.6%)^A^	8 (0.7%)^B^	42 (3.8%)	1,041 (94.7%)
Single EIF (n = 662)	1 (0.2%)	0	0^C^	5 (0.8%)	19 (2.9%)	637 (96.2%)
Multiple EIFs (n = 437)	0	0	7 (1.6%)^D^	3 (0.7%)	23 (5.3%)	404 (92.4%)
Control group (n = 5,616)	110 (2.0%)	32 (0.6%)^E^	32 (0.6%)^F^	72 (1.3%)^G^	211 (3.8%)	5,159 (91.9%)

*EIF* echogenic intracardiac focus, *pCNV* pathogenic copy number variation, *lpCNV* likely pathogenic copy number variation, *VOUS* variants of uncertain significance, *lbCNV* likely benign copy number variation, *bCNV* benign copy number variation.

Chi-squared (X^2^) test was applied to compare CNVs detection rate in the groups, A vs (E + F): P = 0.136, B vs G: P = 0.122, D vs (E + F): P = 0.387. Fisher’s Exact test was applied to compare lpCNV detection rate between C and D groups (P-fisher = 0.002).

**Table 2:** CNV-seq findings of 1,099 fetuses with isolated EIF and follow-up outcome.

Case	GA (weeks)	MA (years)	Ultrasonic manifestation	CNV-seq results	Copy number	Origin	Pathogenicity	The main reasons for pathogenicity classification	Follow-up outcome
EIF208	27.2	29	EIF in left ventricle (single)	Trisomy 21	3	De novo	Pathogenic	Whole chromosome aneuploidy	Pregnancy termination
EIF44	24	26	EIF in left ventricle (multiple)	seq[hg19]del(3)(p26.3p26.2) Chr3:g.320000_2920000del (2.60 Mb)	1	De novo	lpCNV	DGV: not found; ClinGen: haploinsufficiency score of CNTN6 gene and CNTN4 gene was 1; De novo	Normal development at 10 months
EIF361	26.1	33	EIF in both ventricles (multiple)	seq[hg19]del(4)(q12q13.2) Chr4:g.57540000_69300000del (11.76 Mb)	1	De novo	lpCNV	DGV: not foun; ClinGen: haploinsufficiency score of YTHDC1 gene was 1; OMIM: REST is the pathogenic gene of Fibromatosis, gingival, 5, autosomal dominant; Large CNV, cytogenetically visible alterations; De novo	Pregnancy termination
EIF364	24.6	31	EIF in left ventricle (multiple)	seq[hg19]dup(9)(q21.13q21.32) Chr9:g.74900000_86380000dup (11.48 Mb)	3	Unknown	lpCNV	DGV: not found; DECIPHER: 13 patients with 9q21 duplication; Large CNV, cytogenetically visible alterations	Ankyloglossia
EIF575	24.4	25	EIF in left ventricle (multiple)	seq[hg19]del(2)(q31.1q31.1) Chr2:g.174580000_176340000del (1.76 Mb)	1	De novo	lpCNV	DGV: not found; OMIM: CHN1 is the pathogenic gene of Duane retraction syndrome 2, autosomal dominant; CHRNA1 is the pathogenic gene of Myasthenic syndrome, congenital, 1A, slow-channel, autosomal dominant; De novo	Pregnancy termination
EIF636	26.5	25	EIF in left ventricle (multiple)	seq[hg19]del(16)(p13.11p13.11) Chr16:g.15120000_16300000del (1.18 Mb)	1	De novo	lpCNV	DECIPHER: covering the 78.7% genomic region of 16p13.11 recurrent microdeletion, phenotypic variability and incomplete penetrance. De novo	Normal development at 5 months
EIF638	27.5	32	EIF in both ventricles (multiple)	seq[hg19]del(16)(p13.11p12.3) Chr16:g.15500000_18120000del (2.62 Mb)	1	De novo	lpCNV	DECIPHER: covering the 66.0% genomic region of 16p13.11 recurrent microdeletion, phenotypic variability and incomplete penetrance. De novo	Pregnancy termination
EIF827	25.3	30	EIF in both ventricles (multiple)	seq[hg19]del(12)(p13.33p13.33) Chr12:g.1100000_1260000del (0.16 Mb)	1	Unknown	lpCNV	DGV: not found; 12p13.33 microdeletion including ELKS/ERC1 is a locus associated with childhood apraxia of speech (PMID: 22,713,806)	Pregnancy termination
EIF103	26.3	30	EIF in left ventricle (single)	seq[hg19]dup(15)(q23q23) Chr15:g.70600000_71840000dup (1.24 Mb)	3	Unknown	VOUS	DGV: not found: DECIPHER: 1 patient with 15q23 microduplication	Delivered at 34.1 weeks, developmental retardation
EIF176	21.6	26	EIF in left ventricle (single)	seq[hg19]del(10)(q26.3q26.3) Chr10:g.134220000_135440000del (1.22 Mb)	1	De novo	VOUS	DGV: not found; DECIPHER: 11 patients with 10q26.3 microdeletion; De novo	Pregnancy termination
EIF336	27.5	24	EIF in left ventricle (multiple)	seq[hg19]dup(21)(q22.3q22.3) Chr21:g.43640000_44520000dup (0.88 Mb)	3	De novo	VOUS	DGV: not found; DECIPHER: 2 patients with 21q22.3 microduplication; De novo	Normal development at 5 months
EIF585	25.5	23	EIF in left ventricle (single)	seq[hg19]del(4)(q22.1q22.1) Chr4:g.91560000_92260000del (0.70 Mb)	1	Unknown	VOUS	DGV: not found; DECIPHER: 2 patients with 4q22.1 microdeletion	Normal development at 10 months
EIF820	25.2	25	EIF in left ventricle (multiple)	seq[hg19]dup(12)(q24.31q24.31) Chr12:g.123420000_124160000dup (0.74 Mb)	3	De novo	VOUS	DGV: not found; DECIPHER: 7 patients with 12q24.31 microduplication; De novo	Normal development at 5 months
EIF948	30.5	21	EIF in both ventricles (multiple)	seq[hg19]dup(8)(q21.11q21.11) Chr8:g.74300000_76320000dup (2.02 Mb)	3	Unknown	VOUS	DGV: not found; DECIPHER: 2 patients with 8q21.11 microduplication	Normal development at 8 months
EIF971	33.5	22	EIF in left ventricle (single)	seq[hg19]dup(4)(p15.2p15.2) Chr4:g.21780000_23840000dup (2.06 Mb)	3	Unknown	VOUS	DGV: not found; DECIPHER: 7 patients with 4p15.2 microduplication	Normal development at 8 months
EIF1003	27.6	24	EIF in left ventricle (single)	seq[hg19]dup(5)(q23.1q23.1) Chr5:g.119160000_120260000dup (1.10 Mb)	3	Unknown	VOUS	DGV: not found; DECIPHER: 1 patient with 5q23.1 microduplication	Normal development at 5 months

*EIF* echogenic intracardiac focus, *GA* gestational age, *MA* maternal age, *lpCNV* likely pathogenic copy number variation, *VOUS* variants of uncertain significance.

### CNV-seq analysis of the control group samples

The control group contained 5,616 samples; the mean gestational age and maternal age at the time of amniocentesis were 21.6 weeks (range 16.0–35.6) and 27.6 years (range 15.0–34.0), respectively. Furthermore, 64 samples (1.1%) had abnormal segmental aneuploidies, and the CNVs in 72 fetuses (1.3%) were classified as VOUS. Statistical difference was not indicated in the incidence of abnormal segmental aneuploidies between the case group and control group (x^2^ = 2.220, P = 0.136); in addition, statistical difference was not indicated in the VOUS incidence between the two groups (x^2^ = 2.397, P = 0.122) (Table 1).

## Discussion

Chromosomal abnormalities occur in approximately 1 in 150 live births; autosomal trisomies are the most common aneuploidies, and T21 is the most common of these, with a prevalence of approximately 1 in 700–800 live births^27–29^. Most of our study participants received cell-free DNA screening, first-or second-trimester screening before amniocentesis. The detection of whole chromosome aneuploidy in amniotic fluid samples cannot objectively reflect its true incidence. Therefore, we have not compared and analyzed the results of whole chromosome aneuploidy in this study.

In our samples, the majority of EIF were unilateral; among the cardiac chambers affected, the left ventricle was the most frequent (69.4%), which was similar to that reported by other authors^2,30^. Some studies have shown that no chromosomal karyotype abnormalities were found in fetuses with multiple EIFs^31,32^. However, Towner et al. suggested that finding multiple EIFs was a stronger predictor of T21 than that described for a single EIF^33^. Our results indicated that all seven fetuses with abnormal segmental aneuploidies had multiple EIFs, and the incidence of abnormal segmental aneuploidies in multiple EIFs samples was 1.6% (7/437). However, no segmental aneuploidies of definite clinical significance were found in 662 fetuses with a single EIF. Therefore, fetuses with multiple EIFs have a higher incidence of abnormal segmental aneuploidies than those with single EIF (P-fisher = 0.002), i.e., multiple EIFs have more clinical significance than single EIF.

**Figure 1:**
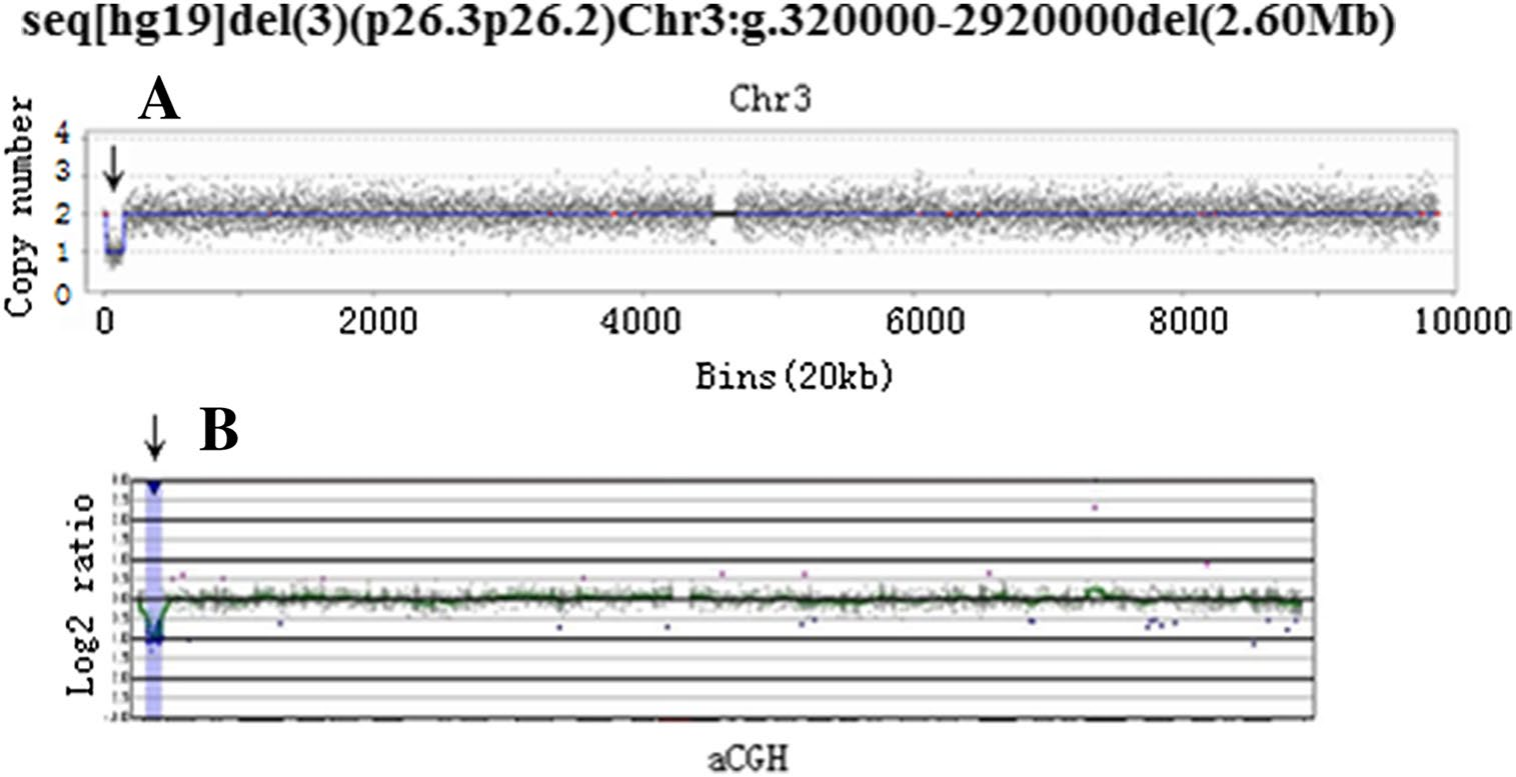
Secondary confirmation of chromosomal abnormalities detected by CNV-seq. CNV-seq profiles of EIF44 (**A**), data are plotted as copy number (Y-axis) versus 20 kb chromosomal read bins (X-axis). The mean copy number along the length of each chromosome is indicated by the blue line. Confirmatory aCGH profiles is shown at the lower part (**B**).

Among the seven cases of lpCNVs, two fetuses were diagnosed with a known syndrome, 16p13.11 recurrent microdeletion, which is a clinically heterogeneous disease; common phenotypes include mental retardation, epilepsy, and multiple congenital anomalies. It appears that this may be a susceptibility locus for neurocognitive disease, where 16p13.11 deletion is insufficient to cause the phenotype^34^. The parents of one fetus (EIF636) decided to continue the pregnancy, and our follow-up data showed that the infant demonstrated normal developmental milestones at 5 months. The parents of the other fetus (EIF638) opted for pregnancy termination. With regard to the other five fetuses with lpCNV: EIF44 (Fig. 1), EIF361, EIF364, EIF575, and EIF827, the follow-up results indicated normal development at 10 months, pregnancy termination, ankyloglossia, pregnancy termination, and pregnancy termination, respectively.

Among the eight samples with VOUS, the parents of only one fetus (EIF176) opted for pregnancy termination. One fetus (EIF103) was delivered at 34.1 weeks and pediatric examinations at 6 months indicated delayed development; however, because the pregnant woman refused to complete a full trio analysis, the origin and pathogenicity of the CNV in this case cannot be identified. Additionally, because of premature birth, we cannot determine the exact cause of the child's growth retardation. The remaining six fetuses were delivered at full term and exhibited normal development within a few months after birth. However, owing to the age of the babies, we could not assess their prognosis fully and accurately; therefore, further follow-up is required.

In our study, apart from whole chromosome aneuploidy, seven cases of abnormal segmental aneuploidies were detected, and the incidence of lpCNVs was 1/157 (7/1,099), which was similar to that reported by other authors^16^. Significant difference was not indicated in the detection rate of abnormal segmental aneuploidie between fetuses with EIF and those with normal ultrasound results (P = 0.136). In addition, although fetuses with multiple EIFs have higher incidence of abnormal segmental aneuploidies than those with single EIF, significant difference was not indicated in the detection rate of abnormal segmental aneuploidie between fetuses with multiple EIFs and those with normal ultrasound results (P = 0.387). Therefore, isolated EIF does not increase the risk of abnormal segmental aneuploidie in the fetus. In clinical work, if a fetus with isolated EIF has no other risk factors leading to segmental aneuploidy, clinicians should theoretically only focus on the risk of the common whole chromosome aneuploidy. Therefore, for fetuses with isolated EIF, if no other high-risk factors exist leading to segmental aneuploidies, screening methods such as cell-free DNA screening or first-or second-trimester screening are recommended to help assess the risk of fetuses suffering from the common whole chromosome aneuploidy; however, interventional prenatal diagnosis is not recommended as the first choice.

Meanwhile, the fragments of five among seven abnormal segmental aneuploidies were less than 5 Mb, which is typically undetectable by karyotype analysis. Combined with previous research data^13,35^, the proportion of submicrostructural abnormalities in fetal chromosomal aberrations is not low. In clinical practice, the presence of soft markers increased the incidence of invasive procedures substantially. Previous studies have shown that in patients at lower levels of T21 risk, the rate of amniocentesis was significantly higher following disclosure of isolated EIF when compared with pregnancies without EIF at similar risk levels^18^. Therefore, if pregnant women with fetal EIF, especially those with multiple EIFs, voluntarily chose invasive prenatal diagnosis, they should be recommended high resolution methods such as CNV-seq or CMA, rather than karyotype analysis.

In conclusion, our data indicated that the incidence of abnormal segmental aneuploidies among fetuses with isolated EIF was 1/157. Isolated EIF did not increase the risk of fetal segmental aneuploidies.

## Materials and methods

### Clinical data

The current study was a prospective case–control study; all pregnant women in the case group and control group were younger than 35 years old at the expected date of childbirth, and all samples were from pregnant women who received prenatal diagnosis in West China Second University Hospital of Sichuan University from February 2017 to December 2018. The study participants in the case group were fetuses that were diagnosed with isolated EIF based on ultrasonogram findings by two experienced ultrasonographers; none of the fetuses indicated any structural abnormalities or other soft markers by ultrasonogram through all the pregnancy stages. Samples without fetal ultrasound abnormalities but received prenatal diagnosis for other reasons (serological screening high-risk, voluntary request) were set as controls. The study was approved by the Medical Ethics Committee of West China Second University Hospital of Sichuan University, and we confirmed that all experiments were performed in accordance with relevant guidelines and regulations. All women underwent genetic counseling and signed an informed consent that explained the possible pathogenic significance of their condition; they voluntarily requested amniocentesis and fetal CNV-seq testing.

### DNA extraction and detection of maternal cell contamination

According to routine operation specifications, 20 ml amniotic fluid was extracted and placed into four sterile centrifuge tubes. CNV-seq and quantitative fluorescence PCR (QF-PCR) were performed on two tubes, and the remaining two tubes were stored at 2–8 °C. DNA was extracted from the amniotic fluid using a DNeasy Blood and Tissue Kit (QIAGEN, Germany), according to the manufacturer’s standard extraction procedures. The quality and concentration of DNA were assessed with the NanoDrop 1000 (Thermo Fisher Scientific, USA). QF-PCR detection was performed using 21 trisomy/sex chromosome/polyploid and 18 trisomy/13 trisomy/polyploid detection kits (DAAN Gene, China), according to the manufacturer’s instructions. If the results of QF-PCR indicated that there were maternal cells in the samples, CNV-seq and QF-PCR were performed on the spare samples after cell culture.

### CNV-seq

The DNA library was obtained using the Chromosome CNV Detection kit (Berry Genomics, China) and was subsequently sequenced on the Illumina Nextseq 500 sequencing platform (Illumina, United States) using the Nextseq 500 High Output kit (Illumina, United States). Finally, we compared the reads obtained by sequencing with the human reference genome and performed bioinformatics analysis to obtain the genomic copy number information of the samples as previously described^14^. In this study, the pathogenicity of CNVs > 100 kb was analyzed. By searching the DGV (https://dgv.tcag.ca/), DECIPHER (https://decipher.sanger.ac.uk/), ClinGen (https://www.clinicalgenome.org/), OMIM (https://omim.org/), and PubMed (https://www.ncbi.nlm.nih.gov/pubmed) databases, the pathogenicity of the CNVs were preliminarily classified into five categories: pCNV, lpCNV, VOUS, likely benign CNV (lbCNV), or benign CNV (bCNV). When lpCNV or VOUS was identified in the amniotic fluid samples, we recommended that the biological parents of the fetus underwent CNV-seq (using peripheral blood samples) to determine the origin of the CNV of the fetus. Then, combined with parents' CNV-seq results and phenotypes, we reclassified the pathogenicity of the CNV. The DNA extraction and CNV-seq methods of the peripheral blood samples were performed as described for the amniotic fluid samples.

### Confirmatory testing of CNVs

Whole chromosome aneuploidies of 13, 18, 21, X, and Y were confirmed by QF-PCR, while pCNV, lpCNV or VOUS were confirmed using array-based comparative genomic hybridization (aCGH) or a second repeat of CNV-seq. aCGH was performed using the CGX v2 Oligo aCGH Kit (Agilent Technologies, USA). The microarray was scanned using the Agilent SureScan Microarray Scanner (Agilent, USA). Data were extracted using the Agilent CytoGenomics sofware (Agilent, USA) and analyzed using the Genoglyphix Analysis sofware (Perkin Elmer, USA)^14^.

### Informed consent

We confirm that informed consent was obtained from all participants and/or their legal guardians.
